# An Integrated Prediction Framework for Engineered Cementitious Composite: EDFrame

**DOI:** 10.3390/ma19122465

**Published:** 2026-06-09

**Authors:** Pan Chen, Yufei Wang, Xin Zhang, Xianda Liu, Han Liu, Qingxiang Zhao, Xiangyu Wang, Wenquan Ni, Shanghua Jia, Huili Wang

**Affiliations:** 1 Shanghai Highway and Bridge (Group) Co., Ltd., Shanghai 200433, China; chenpan_49@sina.com (P.C.); 15679605191@163.com (W.N.); jiashanghuahua@126.com (S.J.); dzh1261743749@outlook.com (H.W.); 2 School of Design and the Built Environment, Curtin University, Perth, WA 6102, Australia; yufei.wang1@postgrad.curtin.edu.au; 3 China Testing & Certification International Group Shanghai Co., Ltd., Shanghai 201203, China; zhangxin@zcet.com.cn; 4 School of Civil Engineering and Architecture, East China Jiaotong University, Nanchang 330013, China; 2024018085901103@ecitu.edu.cn (H.L.); 8686@ecjtu.edu.cn (X.W.); 5 Institute for Smart City of Chongqing University in Liyang, Chongqing University, Changzhou 213300, China; 6 China Construction Fifth Engineering Bureau Co., Ltd., Changsha 410000, China; 15073076011@163.com; 7 National Engineering Technology Research Center for Prefabrication Construction in Civil Engineering, Tongji University, Shanghai 200092, China

**Keywords:** engineered cementitious composite, deep learning, generative adversarial network, model interpretability, visualization

## Abstract

Engineered cementitious composite (ECC) is a high-performance strain-hardening material widely used in durable infrastructure, yet its complex multi-parameter interactions make accurate mixture design and performance prediction challenging. This study aims to establish an EDFrame, which is an integrated prediction framework for engineered cementitious composite (ECC). First, two original datasets of ECC’s tensile stress and strain are collected from the comprehensive and authoritative literature, comprising 18 features and 10 categories of single or hybrid fibers. Data augmentation is then performed using a constraints-modified Conditional Tabular Generative Adversarial Network (Tuned-CTGAN), with two traditional methods for comparison. A One-Dimensional Convolutional Neural Network with a residual module (1D-Residual CNN) is developed to predict tensile stress and strain, and its performance was compared against five popular machine learning models. The interpretability of the proposed model has been achieved through Partial Dependence Plot (PDP) and Kernel SHAP analyses. The results demonstrate that Tuned-CTGAN effectively generates reliable synthetic data, significantly improving the *R*^2^ of 1D-Residual CNN from 0.8658 to 0.9128 for tensile stress and from 0.8433 to 0.9378 for tensile strain, outperforming all compared models. PDP analysis identifies optimal fiber content (1.5–2%) and fiber length (12–20 mm) ranges for enhanced tensile performance, while SHAP analysis reveals fiber length and diameter as the most critical features influencing tensile stress and strain, respectively. The proposed EDFrame provides a robust and interpretable solution for ECC performance prediction, supporting efficient and accurate mixture design in engineering practice.

## 1. Introduction

With the increasing demand for high-performance and durable infrastructure, advanced cementitious materials with superior tensile properties have become a key research focus [1]. However, the complex multi-parameter interactions in engineered cementitious composites (ECC) make efficient and accurate mixture design and performance prediction a significant challenge, highlighting the necessity of data-driven approaches [2,3]. Known as a strain-hardening cementitious material, ECC exhibits high tensile strength and ductility mainly due to the micromechanical reinforcement of the fiber [4]. By homo-dispersing fiber in the composite matrix and limiting the fiber volume below 2%, ECC can be designed to demonstrate strain-hardening behavior with multi-cracks less than 100 μm. The tensile strength of ECC typically ranges from 4 to 20 MPa, and its tensile strain capacity ranges from 3 to 12% [5]. This tensile ductility of ECC surpasses that of plain concrete by two to three orders of magnitude, highlighting its excellent durability in extreme environments and its promising engineering applications.

The application value and research potential of ECC have become a consensus due to its two key characteristics: ultra-high tensile strain capacity and special multi-microcrack pattern. The current investigation encompasses various aspects of ECC, including its design theory, mixture design, tensile properties, crack models, fiber interfacial enhancement, durability, etc., [6,7,8]. These research hotspots revolve around ECC’s high tensile characteristics, serving as a foundation to achieve precise mixture design with multi-objective balance (e.g., cost effectiveness, high tensile property, good durability, and easy implementation).

Apart from the external effects, such as loading modes (static or cyclic), strain rates, and specimen geometry, the mixture ingredients of ECC play a pivotal role in significantly influencing its tensile properties. Notably, the type of fibers used, such as polyethylene fiber (PE), polyvinyl alcohol fiber (PVA), polypropylene fiber (PP), and steel fiber (SF), as well as specific fiber parameters like the nominal strength, diameter, length, Young’s modulus, and oil coating, all exert varying influences on ECC. For instance, PVA-ECC exhibits a maximum tensile strain of 5%, while PE-ECC can even reach 13% without any fiber surface treatment [5,9]. Researchers have also explored the hybrid usage of various types of fibers, aiming to integrate the advantages of different fiber reinforcements into ECC [10]. In addition, the inclusion of mineral admixtures (i.e., cement, fly ash, slag), solid wastes (e.g., waste rubber, glass particles, tailings, etc.), and admixtures (e.g., superplasticizer, viscosity agent, defoamer, etc.) is a vital part of complex mixture design for ECC. Despite a substantial number of published articles up until 2018 (approximately 10,000 relevant ones), most investigations have focused only on specific aspects of the above-mentioned features [5]. This has led to two main issues: (1) the literature review and experiment verification processes become time-consuming and labor-intensive, (2) the design theory and equations based on the partial experimental data may lack accuracy. Therefore, the establishment of an ECC database and the development of a reliable, fast, effective, and time-saving method for ECC design are promising and pressing. Addressing these challenges would greatly benefit the advancement of ECC technology and its practical applications.

Previously, researchers attempted to derive the initial mixture design meeting engineering needs by applying empirical equations and conducting multiple experiments to search for the optimal design scheme [11]. Theory-based design equations or conventional fitting equations, such as linear regression and multi-linear regression, are frequently used to seek the relationship between mixture variables and outputs [12]. However, these attempts often fell short in yielding desirable outcomes, due to the complex microstructural heterogeneity and the complexity of ECC [13]. Recently, the rapid advancement of artificial intelligence (AI) has introduced the machine learning (ML) method, which has found extensive application in concrete properties’ prediction, such as compressive strength, flexural strength, and permeability [14,15]. In the context of ECC, machine learning has attracted growing attention for predicting mechanical performance and assisting material design [16]. Recent international studies have shown that advanced ML techniques, including ensemble learning and deep learning, can effectively capture the complex nonlinear relationships among mixture parameters and significantly improve prediction accuracy for cementitious materials [17,18,19,20]. Furthermore, data-driven approaches have been extended to ECC systems to predict mechanical properties and support multi-objective material design under various constraints [21,22]. Despite these advances, most existing studies still rely on relatively limited datasets and often focus on specific material configurations, such as single fiber types, which restricts their generalization capability [23]. This limitation highlights the need to construct more comprehensive and representative datasets and to introduce advanced data augmentation strategies to improve model robustness and applicability. In addition, commonly used ML models, including artificial neural networks (ANN), support vector regression (SVR), and ensemble learning methods, have demonstrated strong predictive performance in concrete-related applications, further indicating the potential of data-driven approaches for complex material systems [24,25,26].

The advantages of employing ML for characteristics prediction are obvious since it allows for the accurate capture of the nonlinear relationships between features and outputs. However, a significant and inevitable challenge arises from the weak generalization ability of established ML, hindering their universal applicability in designing ECC with different ingredients. This limitation primarily stems from the small database and, consequently, simple ML models, which pose challenges restricting the further development of deep learning (DL) applications in concrete terms. Therefore, few relevant studies can be found in the literature. One such study by Huynh et al. (2020) [27] attempted to use a deep neural network (DNN) and a residual network (ResNet) to predict the compressive strength of fly ash-based geopolymer concrete. However, the limited size of their database, consisting of only hundreds of samples, resulted in their poor generalization ability. Unfortunately, a dedicated database specifically collected for ECC and DL is non-existent. To address this issue, data augmentation based on a small database is promising. In this regard, the Generative Adversarial Network (GAN) emerges as a potential and powerful tool for generating synthetic data [28]. Implementing GAN-based data augmentation could substantially enhance the size and diversity of the database, thus enabling more robust and accurate DL models for ECC characteristics prediction.

GAN specializes in generating new data and simultaneously preserving the distribution characteristics of the raw database based on the theory of a two-player game (discriminator D and the generator G). Although GAN has found wide application in computer vision (CV) for generating images, its usage in augmenting tabular data (e.g., concrete datasets) remains rare [29,30]. One GAN-based model called TableGAN has been designed to synthesize fake tabular data, where the D and G are convolutional neural networks (CNN). Marani, Jamali et al. (2020) [31] successfully combined TableGAN and ML models to predict the compressive strength of ultra-high-performance concrete (UHPC). However, TableGAN exhibited limitations when dealing with highly imbalanced or incomplete datasets, prompting the development of Conditional Tabular GAN (CTGAN) [25]. CTGAN addresses the challenges of data imbalance by applying the variational Gaussian mixture model (VGM) to simulate continuous features and leveraging a conditional generator to evenly sample discrete features, effectively tackling the problems of data imbalance. Therefore, this data augmentation method creates a promising approach for DL applications in improving the generalization ability of ECC properties’ prediction.

To fill the gap of effective integration of big data and deep learning in generalized ECC prediction and subsequent mixture design, this study proposes EDFrame, an integrated framework with distinct novelties. EDFrame (ECC Data-driven Framework) is defined as an integrated data-driven framework that combines data augmentation, deep learning-based prediction, and model interpretability, enabling accurate, robust, and interpretable prediction of ECC tensile properties. First, unlike conventional GAN-based methods such as TableGAN or CTGAN that generate synthetic data without domain constraints, this study introduces a constraints-modified Tuned-CTGAN that incorporates fiber-specific physical boundaries, ensuring that generated fiber parameters conform to realistic material properties. This framework combines Tuned-CTGAN with a One-Dimensional Convolutional Neural Network incorporating a residual module (1D-Residual CNN), enabling robust prediction on augmented datasets. Second, beyond mere prediction accuracy, EDFrame integrates two important interpretability tools—Partial Dependence Plot (PDP) [32] and Kernel SHAP [33]—to provide both global and local interpretability, elucidating the underlying mechanisms linking mixture ingredients to mechanical performance. Collectively, these contributions establish EDFrame as a comprehensive and interpretable framework that advances beyond existing approaches, which typically focus on isolated aspects of prediction.

## 2. Database Description

To achieve the generalized prediction of ECC properties, a comprehensive database was constructed based on both an existing dataset and a structured review of recent studies. The data were collected from representative publications focusing on conventional ECC mixtures with Ordinary Portland cement [1,2,34,35,36,37,38,39], fiber-reinforced systems with varying fiber types and contents [40,41,42,43,44,45,46,47], and mixtures incorporating different admixtures and curing conditions [48,49,50,51,52,53,54,55,56,57,58,59,60,61,62]. Strict selection criteria were applied to ensure data consistency and reliability:

(1) Ordinary Portland cement is used to produce ECC in the references.
(2) The natural aggregate is selected as the fine or coarse aggregate with an appropriate size distribution instead of other types, such as lightweight aggregate.
(3) The references are selected from authoritative international journals to ensure data reliability.
(4) The characteristics of fiber and other admixtures must be clearly stated.

To be specific, the features of the database collected in this study include four main categories of features: main ingredients of mortar normalized by weight sum of 1 (e.g., cement, water, fly ash, etc.), fiber characteristics (e.g., fiber type, volume proportion, diameter, etc.), admixtures (e.g., superplasticizer, viscosity agent, oiling agent, etc.), and experimental conditions (e.g., temperature, water curing days, air curing days, etc.).

The database covers a comprehensive range of fiber reinforcement, including PE, PVA, PP, basalt, steel, ultra-high molecular weight polyethylene fiber (UHMWPE), and hybrid fiber where two types of fiber are combined (i.e., PE-steel, PVA-calcium carbonate whiskers, PVA-steel, and PP-steel). All of the above-selected features have been approved to exert an essential effect on the strength and ductility of ECC [5]. The outputs of the dataset are the peak tensile stress (MPa) and peak tensile strain (%). A statistical analysis of ECC stress and strain datasets is summarized in Table A1 and Table A2.

The compiled dataset comprises 450 tensile stress samples and 423 tensile strain samples extracted from peer-reviewed experimental studies, each characterized by 18 input variables describing mixture proportions, fiber properties, admixtures, and curing conditions. Key parameters span wide intervals, including fiber volume fraction (0.003–0.20), fiber length (2–38 mm), tensile stress (1.8–33.4 MPa), and tensile strain (0.017–17.3%), indicating substantial variability across different ECC formulations. This wide coverage ensures that the dataset reflects diverse material behaviors under varying design conditions and provides a reliable basis for data-driven modeling.

## 3. Methodologies

The methodology employed in this study consists of three progressive parts: ECC database augmentation, DL prediction model development, and model interpretability. The ECC database augmentation is achieved by combining the collected original data mentioned in Section 2 and the unsupervised learning algorithm—Tuned-CTGAN. Additionally, other adjusted augmentation algorithms are adopted for comparison, including the GaussianCopula Model and Triplet-based Variable AutoEncoders (TVAE). As previously mentioned, the primary objective of this part is to create sufficient data on ECC to establish a robust deep learning model, while also ensuring that the database covers the universal ECC mixture design.

The second part involves the development of the prediction model, wherein a modified 1D-Residual CNN is introduced for ECC properties’ prediction using both the original and synthesized databases. Additionally, the traditional and classical ML models are also trained for comparison, comprising Random Forest (RF), Gradient Boosting Regressor, XGB Regressor, Hist Gradient Boosting Regressor, and SVR.

The third part focuses on model interpretability and visualization based on the establishment of a modified 1D-Residual CNN. Since deep learning is perceived as an opaque “black box”, meaning that only the final results (i.e., the inputs and corresponding outputs) can be observed. This limitation creates an obstacle that hinders the link between the DL model and the ECC mechanism. Therefore, to thoroughly investigate the ECC’s mechanics or ductility from the perspective of DL, model interpretability and visualization (locally or globally) are essential. This will also contribute to the comprehension of the underlying processes and outputs of a DL model. Therefore, PDP and Kernal SHAP are introduced to provide global and local interpretation, respectively.

**Figure 1:**
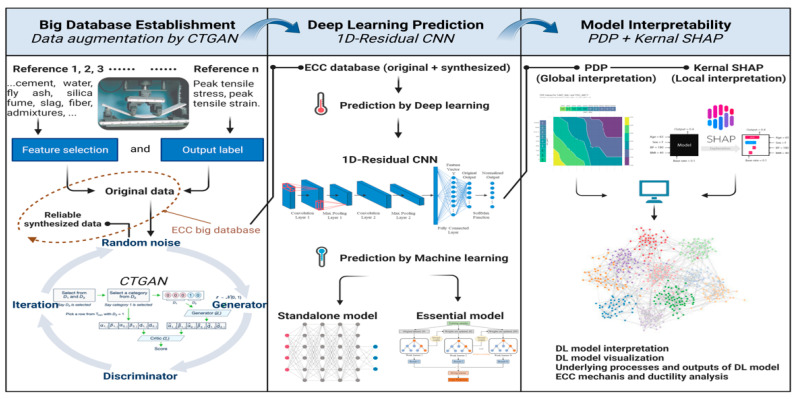
The framework for ECC prediction (EDFrame) comprising data augmentation, DL prediction, and model interpretability.

The above-mentioned framework, referred to as EDFrame, is expected to be valuable in accurately predicting and providing detailed explanations for ECC properties, as exhibited in Figure 1.

### 3.1. Data Augmentation

#### 3.1.1. Concepts of Tuned-CTGAN

Conditional Tabular GAN (CTGAN) is a GAN-based architecture specifically designed for synthesizing tabular data, which was originally introduced by Xu, Skoularidou et al. (2019) [25]. A key challenge in augmenting tabular data augmentation for continuous columns is dealing with non-Gaussian and multimodal distributions, a problem that traditional GAN (e.g., TableGAN) has not effectively addressed. Traditional GANs often use the min-max normalization technique to normalize continuous values to the specific range from −1 to 1. Conversely, CTGAN addresses the issue of non-Gaussian and multimodal distributions by employing a modality-specific normalization technique, transforming continuous values into a bounded vector. This technique involves independently modeling each continuous column using a Variational Gaussian Mixture (VGM) model, expressed as follows:

(1) $$P_{C_{i}}\left(c_{i,j}\right)=\sum_{k=1}^{m_{i}}\;\mu_{k}N\left(c_{i,j};\eta_{k},\phi_{k}\right)$$

where $C_{i}$ represents the ith continuous column of the table, $m_{i}$ denotes the number of modes in the VGM, and $\mu_{k}$, $\eta_{k}$, and $\phi_{k}$ are the weight, mean, and standard deviation of each mode, respectively. For each value $c_{i,j}$ in $C_{i}$, the probability that $c_{i,j}$ originates from each mode in the VGM is calculated as follows:

(2) $$\rho_{k}=\mu_{k}N\left(c_{i,j};\eta_{k},\phi_{k}\right),\;k=1,2,\ldots ,m$$

The mode with the highest probability (assume mode k) is selected, followed by the normalization process, which is represented as follows:

(3) $$\alpha_{i,j}=\frac{c_{i,j}-\eta_{k}}{4\phi_{k}}$$

(4) $$\beta_{i,j}=[0,\ldots ,0,1,0,\ldots ,0]$$

where $\alpha_{i,j}$ is a normalized scalar whose range is constrained within [−1, 1], $\beta_{i,j}$ represents a one-hot encoding with the kth element set to 1 (corresponding to the mode k). Each value of a continuous column is then represented by the combination of a scalar $\alpha_{i,j}$ representing the normalized value and a one-hot vector $\beta_{i,j}$ indicating the mode. This normalized data serves as the input to the model.

During the process of augmenting tabular data for discrete columns, the issue of imbalanced discrete columns in the original database can lead to an imbalance in the discrete value output (mode collapse). To tackle this problem, two techniques are introduced: a conditional generator and sampling training, both aimed at ensuring equal occurrences of all categories in the discrete columns. Specifically, the conditional generator is trained to generate data according to the conditional information of a specific value $k^{*}$ in a specific column $D_{i^{*}}$. To represent this condition, a masking vector $m_{i}$ is introduced as follows:

(5) $$m_{i}^{\left(k\right)}=\{\begin{array}{c} 1\;if\;i\;=\;i*\;and\;k\;=k*\\ 0\;otherwise \end{array}$$

For all discrete columns $D_{1},\;\ldots ,\;D_{N_{d}}$, the cond vector is introduced to concatenate different masking vectors, which is represented as Equation (6).

(6) $$cond=m_{1}⊕m_{2}⊕\cdot \cdot \cdot ⊕m_{N_{d}}$$

The cond vector allows for conditioning specific column values using one-hot encoding. Subsequently, the conditional generator G takes random noise and cond vector as inputs so that the G will be forced to comply with the given condition by reducing conditional loss—cross-entropy.

#### 3.1.2. Establishment of Tuned-CTGAN

The Tuned-CTGAN architecture consists of a conditional generator G and a discriminator D. The generator G is designed with two fully connected layers, which are employed where each is accompanied by a batch normalization layer and a ReLU activation layer. After these two hidden layers, a mixed activation function is used to generate synthetic row representations. Specifically, the values $\alpha_{i}$ in a continuous column, are activated by Tanh, and the values $d_{i}$ in a discrete column, as well as mode indicators$\;\beta_{i}$ are activated by Gumbel Softmax. The embedding_dim, which is a hyperparameter representing the number of random samples received by G, is set to 32 in order to provide variability and richness for the generation process. In addition, the batch size, the number of training times (epochs), and the learning rate are set as 50, 2000, and 2 × 10^−6^, respectively, which are determined according to the training efficiency and time of Tuned-CTGAN.

**Figure 2:**
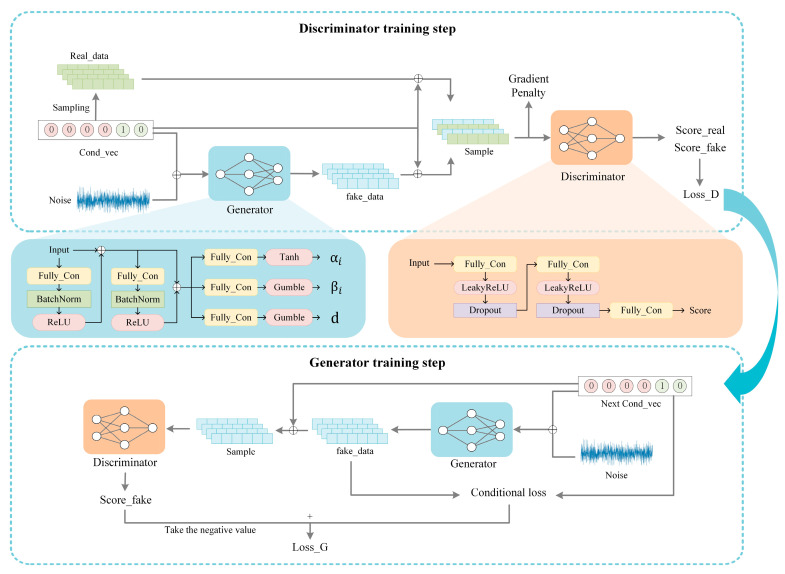
The training process and architecture of the Tuned-CTGAN model.

Within the framework of D, two fully connected layers are employed, followed by a dropout layer to selectively dropout nodes and mitigate overfitting issues. The activation function of LeakyReLU is utilized, and a fully connected layer is subsequently connected to output the score for the current batch. The size of the output samples for each one of the discriminator layers, represented as discriminator_dim, is designed as (512, 512). A large dimension of 512 contributes to accurate differentiation between real and generated data. In addition, the learning rate of the discriminator is set as 0.0005. The architecture and training process of the Tuned-CTGAN are illustrated in Figure 2. The discriminator_steps is three, which represents that three updates of the discriminator will be performed followed by one update of the generator.

It is noted that the fiber parameters should completely match the fiber type during the process of data generation; namely, the tensile strength and elastic modulus of the fiber must conform to the specific fiber type. In this case, the fiber parameters’ lower and upper boundaries were determined based on the original dataset, where the mean value possesses the highest possibility to be chosen. To be specific, the probability of selecting the median is 50%, and the selection probability decreases towards the upper and lower boundaries to zero. This process ascertains the match relationship between specific fiber types and characteristics, increasing the reliability of the fake data generalization. The pseudocode is shown in Algorithm 1.
**Algorithm 1.** Fiber Constraint for Synthetic Data Generation [63]Require: *desired_strength, desired_modulus* 1:   Initialize dictionaries: *desired_strength, desired_modulus* 2:   **function** ISVALID(*column_names, data*) 3:          Extract *fiber_type, fiber_tensile_strength, fiber_elastic_modulus* from data 4:          Initialize *valid_rows* as False array 5:          **for** ft in *desired_strength/modulus*_*dictionaries*() do 6:                 Check if values are within range for strength and modulus 7:                 Update valid_rows using OR operation 8:          **end for** 9:          **return**
*valid_rows* 10: **end function** 11: **function** TRANSFORM*(column_names, data*) 12:         Extract *fiber_type, fiber_tensile_strength, fiber_elastic_modulus* from data 13:         for ft in *desired_strength/modulus_dictionaries*() do 14:                Calculate *avg_strength* and *std_strength* 15:                Generate new strengths using normal distribution 16:                Update *fiber_tensile_strength* for these rows 17:                Calculate avg_modulus and std_modulus 18:                Generate new modulus values using normal distribution 19:                Update *fiber_elastic_modulus* for these rows 20:         **end for** 21:         **return** data 22: **end function** 23: Create custom constraint class using ISVALID and TRANSFORM

#### 3.1.3. Other Data Augmentation Methods

To assess the effectiveness and advancement of the established Tuned-CTGAN, other data augmentation methods are employed for comparison, comprising the GaussianCopula Model [64] and TVAE [25]. The concepts and structures of these two methods will not be specifically discussed in this paper. By comparison, the effectiveness of Tuned-CTGAN in generating realistic and diverse tabular data can be effectively evaluated.

### 3.2. Deep/Machine Learning Model Establishment

#### 3.2.1. DL Model—1D-Residual CNN

The traditional convolutional neural network (CNN) has been proposed to solve various problems, such as computer vision, signal processing, and structural health monitoring [65,66,67]. In this study, an improved 1D-Residual CNN has been proposed to predict the tensile strength and strain of ECC. To facilitate model training and evaluation, the ECC dataset was randomly divided into a training set (80%) and a test set (20%) using the scikit-learn library in Python 3.10. The training set was utilized to train the model, allowing it to learn and optimize its parameters. Meanwhile, the test set was used to evaluate the model’s performance and assess its generalization ability. Before the data augmentation, epochs, batch size, and learning rate were set to 3000, 32, and 5 × 10^−5^, respectively. These parameters were changed to 2000, 256, and 0.00002 after the data augmentation, which were determined to strike a balance between achieving sufficient model convergence and minimizing the risk of overfitting.

**Figure 3:**
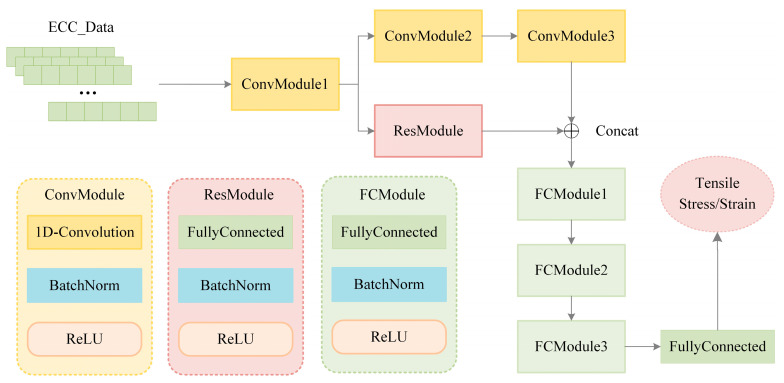
The network structure of 1D-Residual CNN.

The structure of the 1D-Residual CNN consists of three convolutional modules, three fully connected modules, and one residual module, as shown in Figure 3. Each convolutional module consists of a 1D convolutional layer, a batch normalization layer, and the activation function of a Rectified Linear Unit (ReLU). The 1D convolutional layer is employed to extract localized features and capture the inherent dependencies within the input data. The number of convolution kernels is 16, 64, and 32, respectively, and the corresponding kernel sizes are 3, 2, and 1. To enhance model stability and accelerate convergence, batch normalization layers are utilized to normalize the outputs of the convolutional layers. A nonlinear activation function ReLU is finally introduced, allowing the model to adapt to intricate data patterns, enriching the model’s representational capacity.

Similarly, the fully connected module consists of a fully connected layer, a batch normalization layer, and the ReLU activation function. It transforms the outputs from the convolutional layers into the final prediction results through a series of fully connected operations. The number of neurons in each fully connected layer is 416, 256, 64, and 1, respectively.

**Figure 4:**
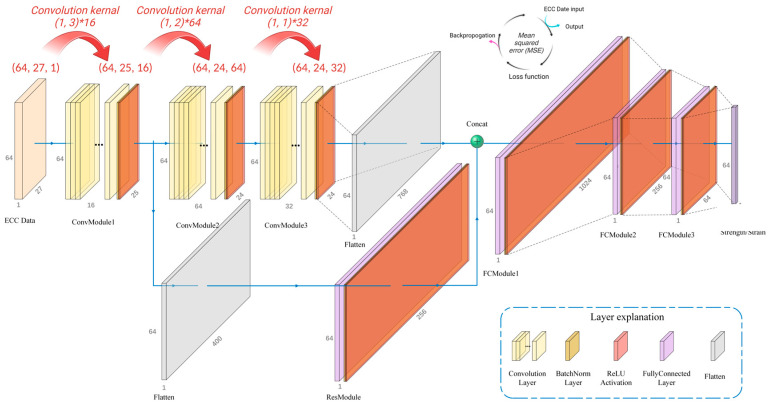
The dimensional variation in tensors in the 1D-Residual CNN.

The residual module shares the same structure as the fully connected module, which is constructed to capture both the low-level and high-level features of input data. By connecting the output of the first convolutional layer to the output of the third convolutional layer, the residual module effectively addresses the issues of gradient vanishing and overfitting. Considering the multiple features in the dataset of ECC, the introduction of the residual module is effective in enhancing the model’s expressive power, prediction accuracy, and generalization capability, and simultaneously increasing the depth of the network without adding parameters. The dimensional variation in tensors in the 1D-Residual CNN is illustrated in Figure 4.

#### 3.2.2. Other ML Models

This paper introduces five ML models for comparison with the 1D-Residual CNN, which are RF, Gradient Boosting Regressor, XGB Regressor, Hist Gradient Boosting Regressor, and SVR [68,69,70]. These classical ML models have shown their good accuracy and generalizability in solving regression problems; they are used for comparison [71,72,73].

### 3.3. Evaluation Index

#### 3.3.1. Evaluation Metrics for GANs

Distinguishing the quality of data generated by GANs is difficult since various evaluation metrics result in different outcomes, making it difficult to draw persuasive conclusions about the model’s performance. To address this issue, this study introduces an integrated method with visual, statistical, and DL/ML-based metrics to evaluate the task of tabular data generation. The visual and statistical-based metrics are described as follows, and the DL/ML-based metrics are specifically illustrated in Section 4.2.

(1) Visual-based metrics: Visualizing the synthesized data allows for an intuitive observation of the similarity between real and synthetic data. This study introduces three visualization evaluation methods: the distribution plot, the cumulative sum, and the correlation table. The distribution plot visualizes the statistical properties of real and synthetic data, checking the similarity between the two datasets. Similarly, the cumulative sum provides a visual comparison of the distributions per column to evaluate the similarity between the real and generated data. The correlation table evaluates the association between each column of the table, assessing the generator’s performance in accurately modeling the relationships between columns.
(2) Statistically based metrics: This study employs two statistical tests for evaluation, which are the KSTest and CSTest [74]. The KSTest applies the empirical Cumulative Distributed Function (CDF) and two-sample Kolmogorov–Smirnov test to examine the distributions of continuous features. The outcomes delineate the maximal deviation between the observed and expected values of the CDF. Conversely, the CSTest is used to evaluate distributions of discrete columns by conducting a Chi-squared test. The *p* value yield from CSTest indicates the probability that values drawn from the two distinct columns are sampled from the same distribution.

#### 3.3.2. Evaluation Metrics for DL/ML Models

To measure the performance of established ML/DL predictive models, three statistical metrics are presented: Mean Squared Error (MSE), Mean Absolute Error (MAE), and Coefficient of Determination ($R^{2}$), as shown in Equations (7)–(9). MSE measures the overall magnitude of the differences between the predicted and actual values. The measure $R^{2}$ determines the proportion of the variance in the dependent variable (actual values) that is predictable from the independent variable (predicted values). The higher value of $R^{2}$ indicates the better predictive performance of the model.

(7) $$MSE=\frac{\sum_{i=1}^{N}\;{\left(Y_{i}-{\hat{Y}}_{i}\right)}^{2}}{N}$$

(8) $$MAE=\frac{\sum_{i=1}^{N}\;{(Y}_{i}-{\hat{Y}}_{i})}{N}$$

(9) $$R^{2}=1-\frac{\sum_{i=1}^{N}\;{\left(Y_{i}-{\hat{Y}}_{i}\right)}^{2}}{\sum_{i=1}^{N}\;{\left(Y_{i}-\bar{Y}\right)}^{2}}$$

where $Y_{i}$ and $\hat{Y_{i}}$ are the tested and predicted values, respectively; $\bar{Y}$ is the mean value of all the tested values; N is the total number of samples in the test.

### 3.4. Model Interpretability and Visualization

#### 3.4.1. Partial Dependence Plot (PDP)

Partial Dependence Plot (PDP) is one of the global interpretable methods, which represents the impact of one or two features on the DL model’s output [32]. It can provide a linear, monotonic, or complex relationship between the output and varying features. The partial dependency function of regression is shown in Equation (10).

(10) $$f_{xs}\left(x_{s}\right)=E_{xs}\left[f_{xs}\left(x_{s},\;x_{c}\right)\right]=\int f_{xs}\left(x_{s},\;x_{c}\right)dP(x_{c})$$

where $x_{s}$ is the feature and the partial dependency function, f is the established DL model, and $x_{c}$ is the other features in f. The feature (s) are selected features in the study. The feature vectors $x_{s}$ and $x_{c}$ define the whole feature space x. The link between the features in set C and the model output can be established by marginalizing the model output on the feature distribution in set C. A function that solely depends on the features in set S can be obtained by marginalizing other features.

#### 3.4.2. Kernal Shapley Additive exPlanations (Kernal SHAP)

Different from the general behavior explanation provided by PDP analysis, Shapley Additive exPlanations (SHAP) attempts to interpret the individual predictions, assigning each feature an importance value (SHAP value) for a particular prediction [33]. The SHAP value for an individual feature indicates its respective contribution to the overall prediction outcome, elucidating the distinction between the average model prediction and the actual prediction made on the database.

SHAP is a game theory-based local explanation framework, which has the following three properties: (1) local accuracy—it necessitates the explanation model to exhibit the fidelity that is comparable to the original DL model’s output; (2) missingness—it requires that the absence or omission of features within the original input exert no influence on the explanation model’s outcome; (3) consistency—it demands that when the model undergoes modifications that amplify its reliance on a particular feature, the corresponding feature importance assigned by the explanation model should not diminish, irrespective of the presence or absence of other features.

The SHAP framework introduces Kernel SHAP as a model-agnostic approach to approximate SHAP values. Kernel SHAP combines linear Local Interpretable Model-agnostic Explanations (LIME) with Shapley values to construct a localized explanatory model, which is more accurate than other sampling-based estimates [75]. It can be represented as follows:

(11) $$f\left(x\right)=g\left(z’\right)=\varphi_{0}+\sum_{j=1}^{M}\varphi_{j}{z’}_{j}$$

where f is the original DL model, g is the explanation model, z is the simplified input, $x=h_{x}(z)$ is a mapping function to the original method, $\varphi_{0}=f(h_{x}(0))$ represents the model output without all of the simplified inputs.

## 4. Results and Discussion

### 4.1. Data Augmentation

**Figure 5:**
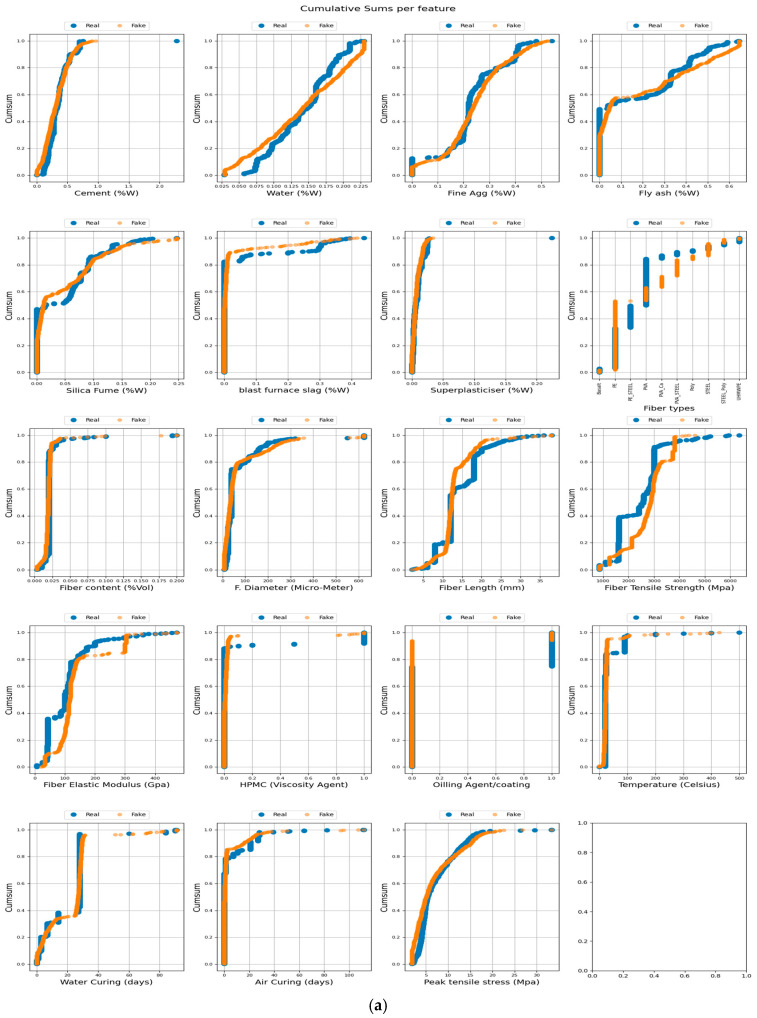

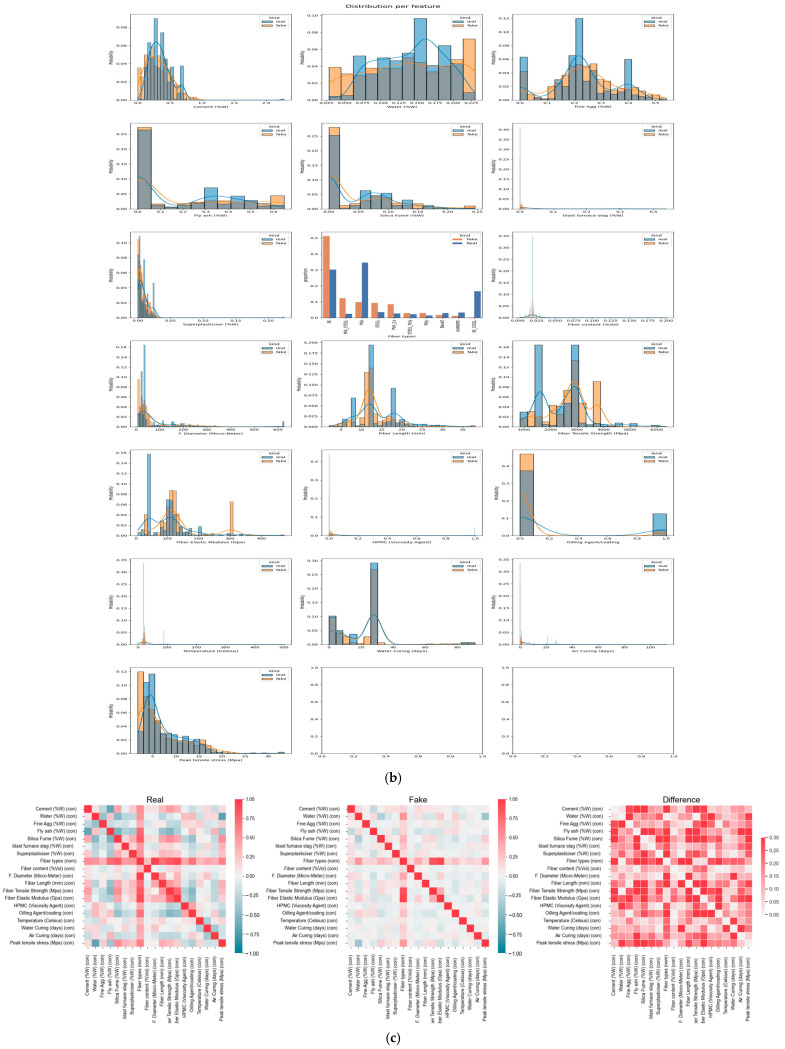
The cumulative (**a**), distribution (**b**), and correlation plots (**c**) for eighteen features before and after Tuned-CTGAN augmentation based on the dataset of tensile stress [63].

**Figure 6:**
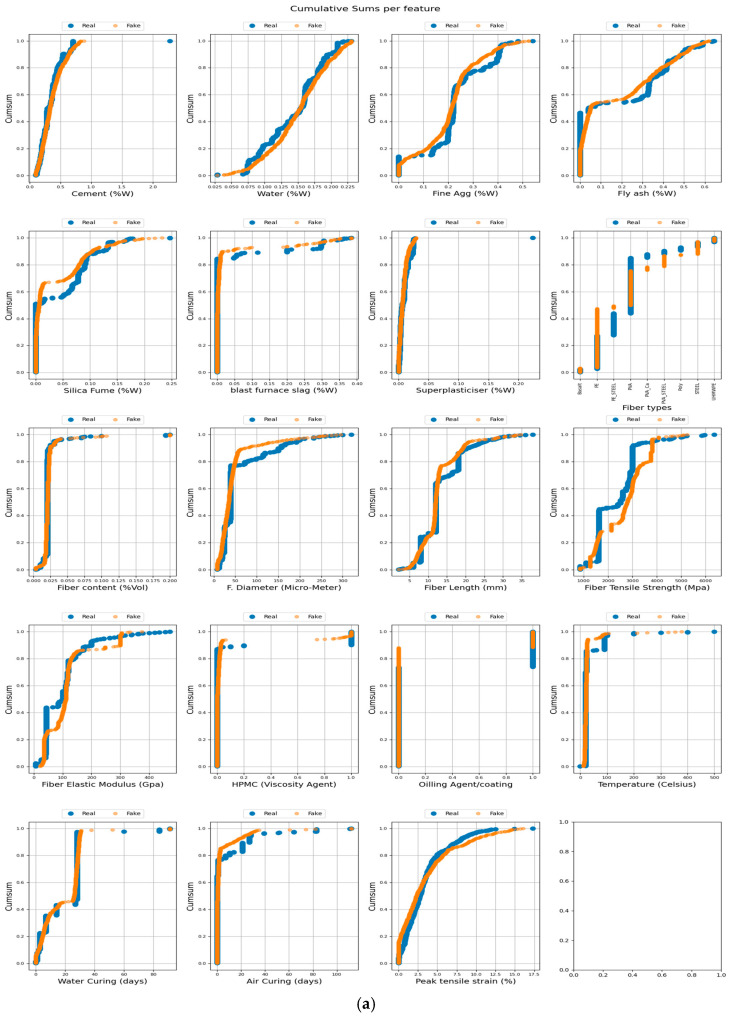

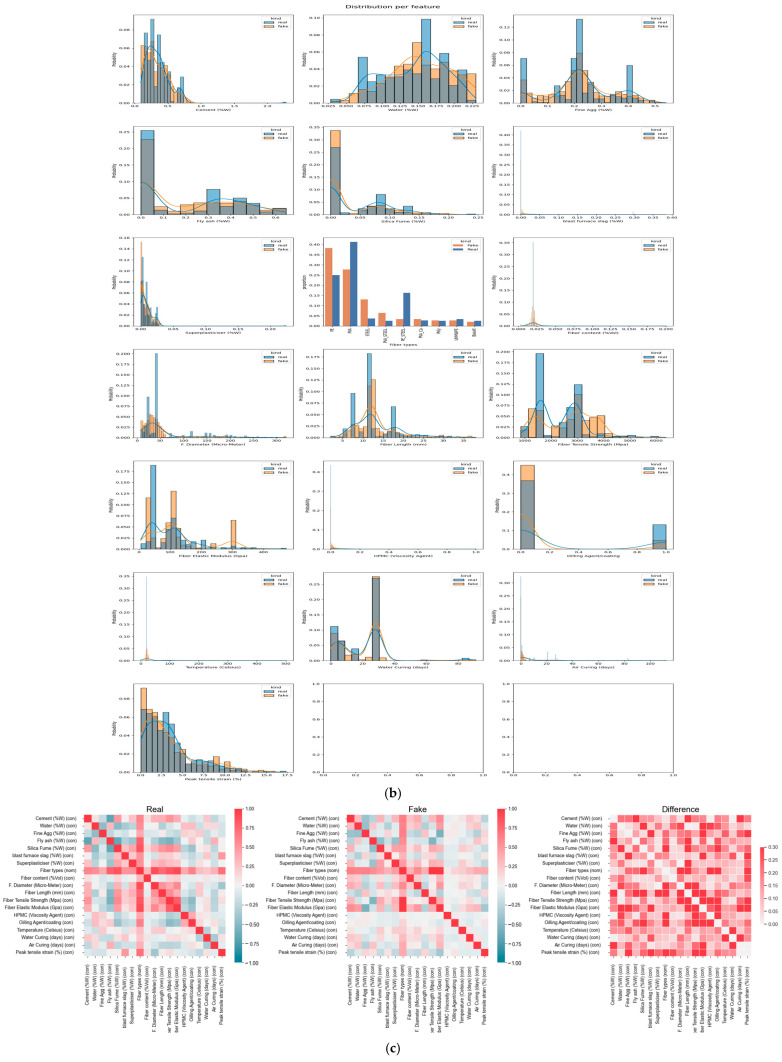
The cumulative (**a**), distribution (**b**), and correlation plots (**c**) for eighteen features before and after Tuned-CTGAN augmentation based on the dataset of tensile strain [63].

The Tuned-CTGAN augmentation technique was used to increase the size of the original tensile stress and strain datasets from 450 and 423 data records to 10,000 each. The performance of the Tuned-CTGAN augmentation on the tensile stress and strain databases is depicted in Figure 5 and Figure 6, respectively, through three visualization plots: the distribution plot, cumulative sum plot, and correlation table. The cumulative and distribution plots show a high similarity for most features between the original and fake data. The distribution of the fiber type (the single discrete variable) was well simulated by the Tuned-CTGAN model, as was clearly shown in the distribution plot. Specifically, the PE and PVA are the two most common categories in the original database, and this is also reflected in the generated fake dataset. However, the distribution of the fiber types was not strictly identical to the original database, mainly due to the characteristics of Tuned-CTGAN. As mentioned earlier, a conditional generator and sampling training applied in Tuned-CTGAN were used to tackle the problem of mode collapse. This technology simultaneously partly neglects the actual distribution of discrete features, increasing the sampling randomness and ultimately leading to no identical distribution. Additionally, the distribution plot and cumulative sum of the other fiber type-related variables, comprising fiber diameter, length, tensile strength, and elastic modulus, were mainly determined by the fiber type. This led to the same phenomenon of no identical distribution as that of fiber type. On the other hand, the distributions related to binder compositions (i.e., cement, water, fly ash, etc.) and environmental factors were similar between real and fake data. The correlation table evaluates the association between each column of the table. In Figure 5c and Figure 6c, the correlation metrics depict a similar pattern with a relatively minor difference (the maximum of 0.3) for both the tensile stress and strain databases. Therefore, the generator’s performance in accurately modeling the relationships between columns has been assessed according to the visualization process.

To explicitly clarify both the dataset scale and distribution, the original tensile stress and strain datasets, consisting of 450 and 423 samples, were expanded to 10,000 samples each after data augmentation using Tuned-CTGAN. Overall, the augmented dataset preserves the main statistical characteristics of the original data while significantly increasing sample diversity. The distributions of most continuous variables show high consistency between real and synthetic data, whereas slight deviations are observed in discrete features due to the conditional sampling mechanism. Nevertheless, the correlation structure and overall trends remain well-maintained, indicating that the augmented dataset provides a reasonable approximation of the original data distribution.

It should be noted that the distribution of discrete features (e.g., fiber type) is not strictly preserved by Tuned-CTGAN. This is mainly due to the conditional sampling strategy, which improves category balance but may slightly deviate from the original frequency distribution. Similar behavior has been reported in tabular GAN studies [26,76], where discrete variables are more difficult to model than continuous ones.

Nevertheless, this deviation has a limited impact on the overall results. In addition to statistical similarity, the physical plausibility of the generated samples is supported through multiple aspects. First, domain-specific constraints are incorporated during data generation to ensure consistency between fiber types and their corresponding mechanical properties. Second, the correlation structure between variables is well preserved after augmentation, indicating that the intrinsic relationships among mixture components remain realistic. Furthermore, the PDP and SHAP analyses reveal physically consistent trends—such as the beneficial role of appropriate fiber content and the influence of matrix composition on strength development—which are consistent with established micromechanical principles of cementitious composites. These observations collectively support the physical realism of the generated data.

The statistically based metrics (e.g., KSTest and CSTest) of the three data augmentation methods for both tensile stress and strain datasets are depicted in Table 1. The KSTest examines the distributions of continuous features, while the CSTest is used to evaluate distributions of discrete columns. For the KSTest, the results are expressed as 1−calculated value, so that a result closer to 1 indicates a slighter discrepancy between the real and generated distributions. In Table 1, the KSTest values of most variables in both stress and strain datasets were over 0.7, indicating a relatively reliable outcome using Tuned-CTGAN data augmentation. The average KSTest values of 18 continuous variables and one output were 0.7784 and 0.7908 on stress and strain datasets, respectively. Meanwhile, the CSTest values of a discrete variable, namely fiber type, were 0.7108 and 0.7144 on stress and strain datasets, respectively. These results explained the reliable data augmentation performance of Tuned-CTGAN, further verifying the conclusions acquired from the visualization plots.

**Figure 7:**
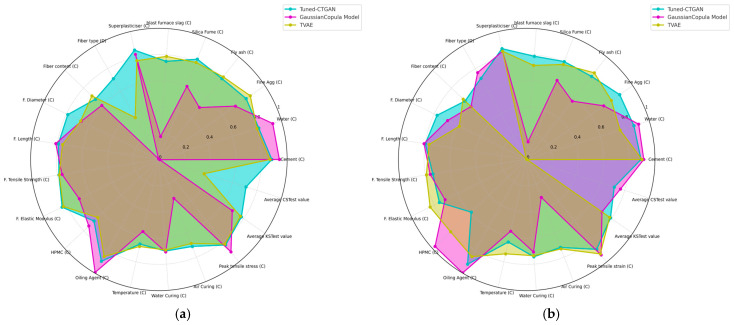
The radar maps of three data augmentation methods on (**a**) tensile stress and (**b**) tensile strain datasets.

To demonstrate the advancement of the proposed Tuned-CTGAN model, the GaussianCopula Model and TVAE were introduced for data generation, with the results shown in Table 1 and Figure 7. Overall, both models showed good performance since the average KSTest values were both around 0.7. However, these values were all lower than those of Tuned-CTGAN, illustrating the worse capability in simulating the variables’ distributions. Moreover, compared to Tuned-CTGAN, the GaussianCopula Model and TVAE exhibited poor performance in simulating fiber type distribution in tensile stress and tensile strain datasets, respectively, with CSTest values of 0.0068 and 0.0031. This could be attributed to the conditional generator and sampling training in CTGAN, together with the specifically proposed constraints in Tune-CTGAN, enhancing the model’s generalizability. Therefore, the usage of both the GaussianCopula Model and TVAE in this task was unreasonable; instead, the virtual data generated by Tuned-CTGAN was used for ML and DL models’ training.

### 4.2. Model Prediction

#### 4.2.1. Prediction Before Data Augmentation

To clarify the data augmentation procedure, the original dataset was first divided into training and test sets. The Tuned-CTGAN was then trained based on the training set, and synthetic samples were generated accordingly to expand the training data. The test set was not involved in the augmentation process and was only used for performance evaluation. The observed improvement in R^2^ is therefore mainly attributed to the increased diversity of the training data after augmentation, which enhances the model’s ability to capture complex relationships between input features and target variables. Otherwise, the model assessment is conducted using a single 80/20 split to ensure a consistent comparison before and after data augmentation. Although more comprehensive validation strategies, such as cross-validation or external datasets, are not considered due to data availability constraints, the use of multiple evaluation metrics and comparative analyses provides a reasonable assessment of model performance.

**Figure 8:**
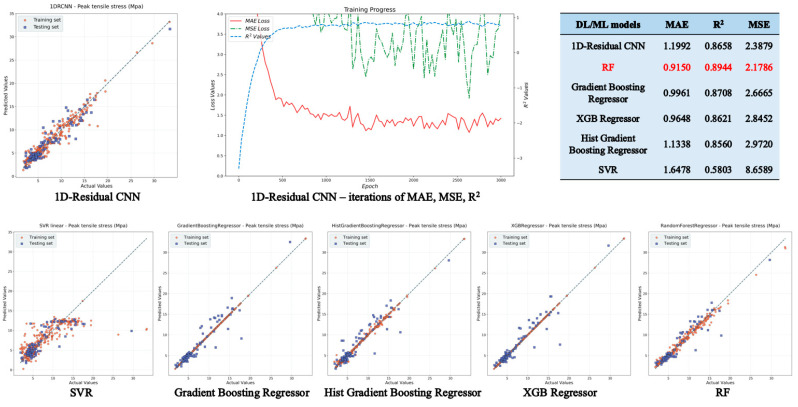
Prediction results by ML/DL models based on tensile stress dataset before data augmentation.

Figure 8 shows the prediction results of the 1D-Residual CNN and five ML methods before data augmentation. The line plots compare the real and forecasted tensile stress of ECC, with the data points’ proximity to the diagonal line indicating the forecast accuracy. A point closer to the diagonal suggests a smaller deviation between observed and predicted values, confirming the forecasting model’s precision.

All models except SVR achieved high prediction accuracy for ECC’s tensile stress, with the R^2^ values of 0.8658, 0.8944, 0.8708, 0.8621, 0.8560, and 0.5803, respectively. Additionally, the relationship between the real and predicted values closely mirrored the linear function y = x. This alignment was particularly evident in the training set since the red data points closely aligned with the diagonal, while the test set displayed a marginally increased dispersion. Combined with the results of MAE and MSE, the RF based on decision trees illustrated the highest prediction accuracy with respective MAE, $R^{2}$, and MSE values of 0.9150, 0.8944, and 2.1786. In contrast, 1D-Residual CNN showed a slightly worse performance in predicting the tensile stress. This can be witnessed in the iteration plots that the MAE, $R^{2}$, and MSE values of D-Residual CNN maintained stability after 1000 iterations, indicating that the model had achieved the optimal weights and bias. This can be attributed to the relatively larger DL model structure than ML models, so it failed to achieve excellent performance in the original small dataset. Additionally, the Gradient Boosting Regressor, XGB Regressor, and Hist Gradient Boosting Regressor showed similar prediction results with roughly the same statistical metrics.

**Figure 9:**
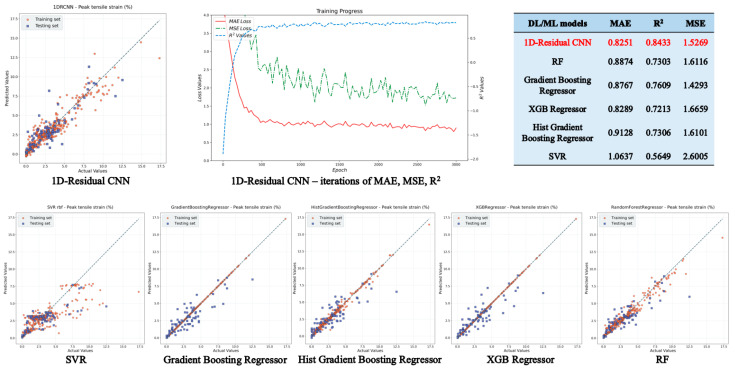
Prediction results by ML/DL models based on tensile strain dataset before data augmentation.

Figure 9 shows that 1D-Residual CNN and ML models performed worse on ECC tensile strain prediction than on stress prediction. Specifically, the R^2^ score for 1D-Residual CNN was 0.8433, while the other five ML models had scores below 0.8, indicating that 1D-Residual CNN had the best forecasting performance. Additionally, the high R^2^ scores for both tensile stress and strain datasets demonstrate that 1D-Residual CNN is more universally applicable than traditional ML models. Furthermore, SVR, the worst model in this task, had the lowest R^2^ score and the highest MAE and MSE scores. In conclusion, RF and 1D-Residual CNN were the two optimal models, exhibiting the best forecast accuracy on ECC tensile stress and strain datasets, respectively.

#### 4.2.2. Prediction After Data Augmentation

**Figure 10:**
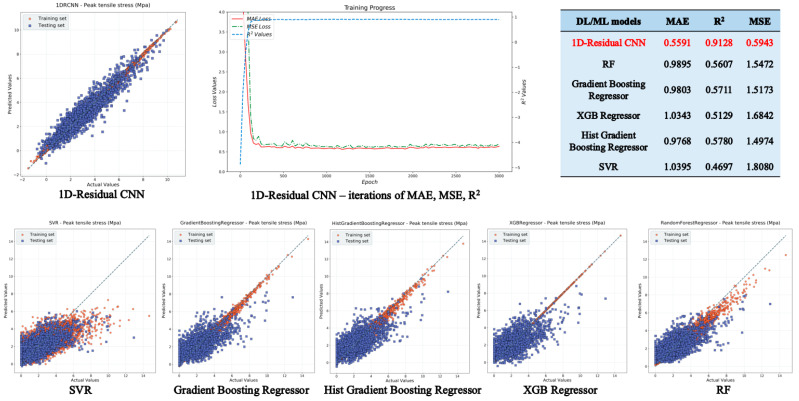
Prediction results by ML/DL models based on tensile stress dataset after data augmentation.

**Figure 11:**
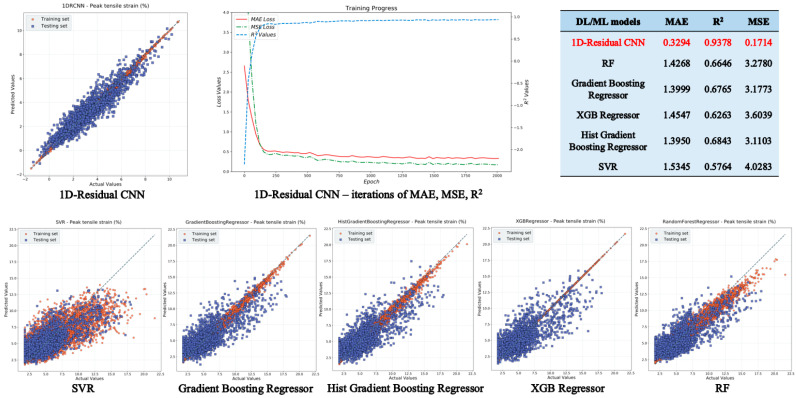
Prediction results by ML/DL models based on tensile strain dataset after data augmentation.

Data augmentation with Tuned-CTGAN increased the number of tensile stress and strain data points to 10,000. Figure 10 and Figure 11 exhibit the forecasting outcome of the 1D-Residual CNN and five ML models on the tensile stress and strain datasets, respectively. The blue data points, representing the test set, closely fit the diagonal line in the scatter plot of 1D-Residual CNN. Meanwhile, the $R^{2}$ of the 1D-Residual CNN on stress and strain datasets increased from 0.8658 and 0.8433 (before data augmentation) to 0.9128 and 0.9378 (after augmentation), respectively. The high increase demonstrates the efficacy of data augmentation in enhancing the prediction performance of the 1D-Residual CNN. This finding was also verified in the decreased values of MAE and MSE.

Compared to the deep learning model, the traditional ML models showed reduced accuracy after the data augmentation, as indicated by the dramatically decreased $R^{2}$ values. Specifically, the $R^{2}$ values of the RF model after the augmentation decreased from 0.8944 to 0.5607 and from 0.7303 to 0.6646 on the stress and strain datasets, respectively. A similar phenomenon was observed in the prediction outcomes of the Gradient Boosting Regressor, XGB Regressor, Hist Gradient Boosting Regressor, and SVR. This indicates that the ML models fail to perform well in solving tasks with big data. In conclusion, data augmentation with Tuned-CTGAN significantly enhanced the predicting performance of the 1D-Residual CNN, while dramatically decreasing that of ML models. The established 1D-Residual CNN was used to perform PDP and SHAP analysis in the next chapter.

It should also be emphasized that the reliability of predictions may decrease in extreme cases, particularly for samples located in low-density regions of the dataset [77]. This is because GAN-based models tend to learn dominant data patterns and may not fully capture tail distributions. Although the overall prediction accuracy is improved after augmentation, caution is required when interpreting results outside the main data range. Future work may incorporate uncertainty estimation or out-of-distribution detection to further enhance robustness.

### 4.3. Model Interpretability by PDP and Kernal SHAP

#### 4.3.1. PDP

**Figure 12:**
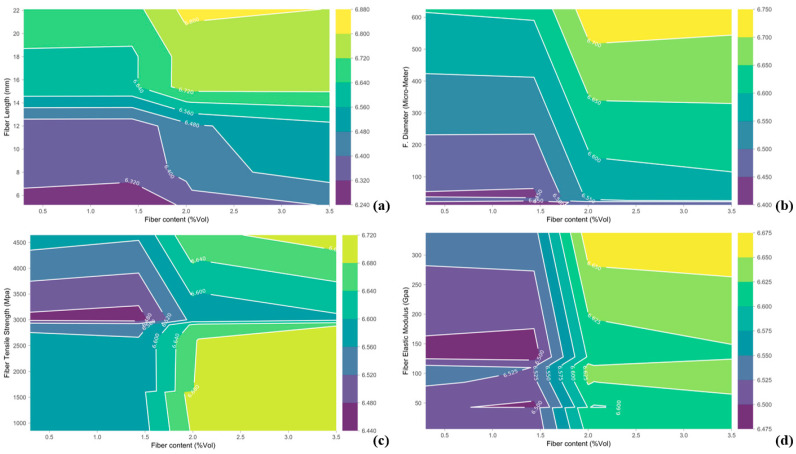
Visualization of predicted ECC’s tensile stress (MPa) using PDP: (**a**) fiber content vs. fiber length; (**b**) fiber content vs. fiber diameter; (**c**) fiber content vs. fiber tensile strength; (**d**) fiber content vs. fiber elastic modulus.

Figure 12 shows the predicted tensile stress (MPa) of ECC against two fiber parameters using PDP based on data augmented with the 1D-Residual CNN. The fiber type, which is a discrete variable, cannot be visualized in this plot. Instead, the fiber parameters comprising fiber length, diameter, tensile strength, and elastic modulus were used to partly represent the fiber type and show their relationships with ECC’s tensile stress.

An obvious positive correlation was observed between the fiber content and tensile stress, since the stress was continuously enhanced with the increase in fiber volume content from 0 to 3.5%. Within a fiber content range of 1.5% to 2%, the contour density exhibited a noticeable increase compared to content levels below 1.5% and those exceeding 2%. This indicates that the optimal fiber content lies within the range of 1.5% to 2%. Both excessive and insufficient levels of fiber content appear to compromise the enhancement of tensile stress. From a materials engineering perspective, this behavior can be attributed to the balance between fiber bridging capacity and matrix workability. An appropriate fiber content enhances crack-bridging efficiency and promotes multiple microcracking, whereas excessive fiber addition may lead to fiber clustering and increased porosity, thereby weakening the matrix integrity. This observation is consistent with the micromechanical design principles of ECC, where optimal fiber dispersion and interfacial bonding are critical for achieving strain-hardening behavior.

As the length of the fibers increases, the interfacial bridging stress exerted by the fibers intensifies. When the fiber content is less than 1.5%, the limited bridging stress between the sparse fibers and the mortar matrix is insufficient to meet the conditions for multi-crack propagation. In cases where the tensile stress over a specified gauge length at the interface is lower than the cracking strength of the matrix, it can lead to a decreased number of interfaces unfavorable for crack propagation. Conversely, the workability can be compromised when the fiber content surpasses the threshold of 2%, leading to defects such as fiber agglomeration and an increased presence of large voids, which will severely undermine the tensile strength of the composite. The influence of fiber length and diameter is closely related to the interfacial bond behavior and pull-out resistance between fiber and matrix. Longer fibers provide greater embedded length, enhancing energy dissipation during crack propagation, while finer fibers contribute to more uniform stress transfer and crack distribution. These mechanisms are fundamental to the strain-hardening response of ECC materials.

Similarly, a sharp increase in tensile stress was witnessed in Figure 12a, with a threshold of around 12 mm of fiber length. This can be attributed to the specific fiber type, according to the original database. Specifically, the length of the PP fiber did not exceed 12 mm, while the lengths of UHMWPE and hybrid fiber (PP-steel) were both over 12 mm. As a result, PP fiber was less effective at enhancing ECC’s tensile stress than UHMWPE and PP-steel. Overall, the effects of fiber length, diameter, and elastic modulus on tensile stress are relatively minor compared to the influence exerted by fiber content. A positive correlation between the fiber diameter and tensile stress. Additionally, Figure 12c shows that fiber tensile strength is positively correlated to ECC’s tensile stress, although some fluctuations were observed. Figure 12d shows that fiber elastic modulus has a minor effect on stress, with some fluctuations. Therefore, fibers with higher fiber tensile strength and diameter are recommended, regardless of fiber type and fiber content.

**Figure 13:**
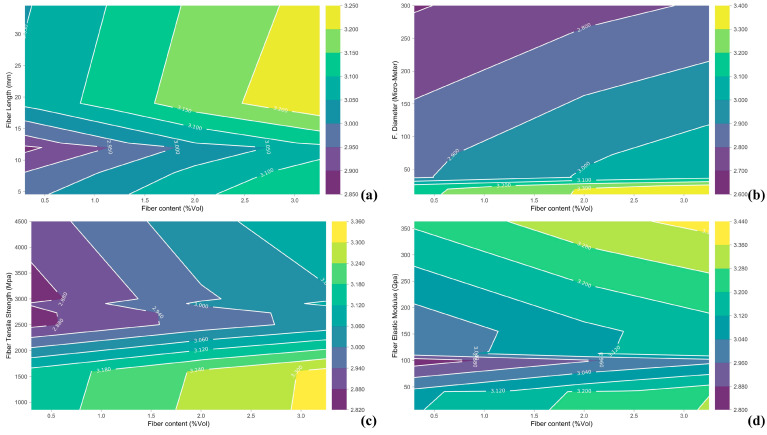
Visualization of predicted ECC’s tensile strain (%) using PDP: (**a**) fiber content vs. fiber length; (**b**) fiber content vs. fiber diameter; (**c**) fiber content vs. fiber tensile strength; (**d**) fiber content vs. fiber elastic modulus.

The PDPs of predicted ECC’s tensile strain are depicted in Figure 13. The relationship between strain and fiber content was similar to that observed in Figure 12, but no obvious dense contours were observed on the horizontal axis, indicating that the tensile strain was continuously improved by the increase in fiber content. In Figure 13a, a positive relationship between strain and fiber length was observed within a range of 12 mm to 20 mm, compared to length levels below 12 mm and those exceeding 20 mm. This suggests a suitable fiber length range for effectively improving strain. Conversely, the fiber diameter had a negative correlation with strain, with fibers below 50 micro-meters in diameter having the highest tensile strain, as shown in Figure 13b. This demonstrates that basalt, PE, PP, PVA, PVA-calcium carbonate whiskers, and UHMWPE tend to possess higher efficacy in improving the ECC’s strain performance than other fiber types. In addition, it can be observed in Figure 13c that fibers with tensile strength values below 2500 MPa yielded the highest matrix tensile strain, challenging the hypothesis that greater fiber tensile strength enhances ECC’s tensile strain. In Figure 13d, the fibers with an elastic modulus over 300 GPa (i.e., PVA-calcium carbonate whiskers, PE-steel) and below 70 GPa (i.e., PVA, PP, PE) corresponded to the highest tensile strain of ECC. This is consistent with the consensus that PVA, PP, and PE are the three most popular fibers used in ECC. Additionally, the hybrid fiber is an effective approach to obtaining fibers with a higher elastic modulus.

#### 4.3.2. Kernal SHAP

**Figure 14:**
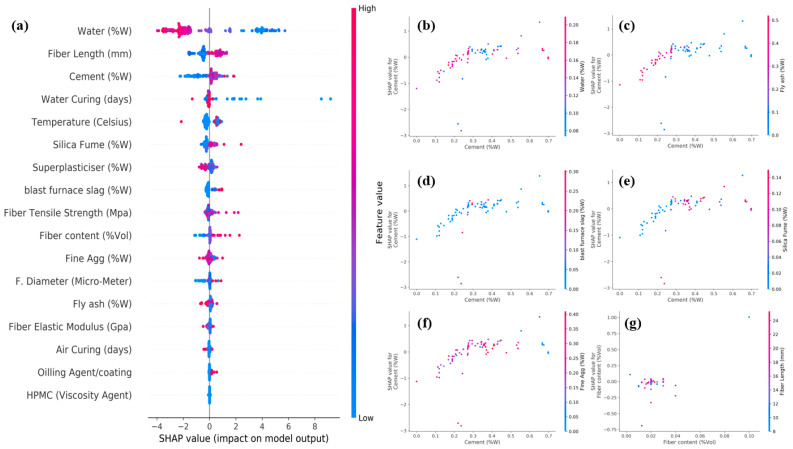
The SHAP analysis showing the influence of each continuous feature on ECC’s tensile stress: (**a**) global feature importance; (**b**) cement SHAP vs. water content; (**c**) cement SHAP vs. fly ash; (**d**) cement SHAP vs. blast furnace slag; (**e**) cement SHAP vs. silica fume; (**f**) cement SHAP vs. fine aggregate; (**g**) interaction of fiber content and length.

Figure 14 shows the negative and positive influence of each continuous feature on ECC’s tensile stress using Kernal SHAP analysis, which uses the Shapley value to explain this influence on a global scale. In Figure 14a, the five most prominent features based on Shapley values were water, fiber length, cement, silica fume, and superplasticizer, in addition to curing conditions. This is reasonable because water, cement, and silica fume components of the binder body significantly determine the matrix’s mechanical performance.

Fiber length was observed as the most important fiber feature for tensile stress due to its bonding, overlap, and bridging role in connecting the mortar matrix. As the fiber length increases, the embedded length at the crack interface serves a greater bridging role, which directly correlates with the interfacial tensile strength. Finally, superplasticizer, a pivotal parameter, influences the workability of fresh ECC material, which ultimately affects the strength of the hardened matrix and the dispersion of fibers within the composite.

In Figure 14b–f, the cement content is plotted on the x-axis to quantify its individual Shapley value, with the point color representing the values of water, fly ash, blast furnace slag, silica fume, and fine aggregate. The cement exhibited a clear positive relationship with the ECC’s tensile stress. Meanwhile, the Shapley value was positive when the cement content (%wt) was greater than 0.3.

As shown in Figure 14b, an increased water content is associated with a decreased Shapley value, evidenced by the predominance of red points below the blue ones. A similar trend was observed for blast furnace slag and silica fume, which were both positively correlated with the Shapley value of cement, illustrating their positive interactive effect on the ECC’s tensile stress (Figure 14d,e). In contrast, fly ash and fine aggregate both showed a minor influence (Figure 14c,f). These findings can be further interpreted from the perspective of matrix densification and interfacial transition zone (ITZ) properties. A lower water content and appropriate cementitious composition contribute to a denser microstructure, improving the fiber–matrix bond strength and delaying crack localization. This highlights the coupled effect of matrix composition and fiber reinforcement in governing ECC tensile performance.

It is important to note that fly ash and fine aggregates have a significant influence on low-strength ECC. Therefore, such ECC types are sensitive to matrix strength and the size of fine aggregates, given the broad range of ECC tensile stress in this study (1.8–33.4 MPa).

Additionally, the increasing Shapley value of fiber content with increasing fiber length is consistent with the findings derived from PDP analysis, further verifying the beneficial hybrid effect of fiber length and content on ECC’s tensile stress (Figure 14g).

**Figure 15:**
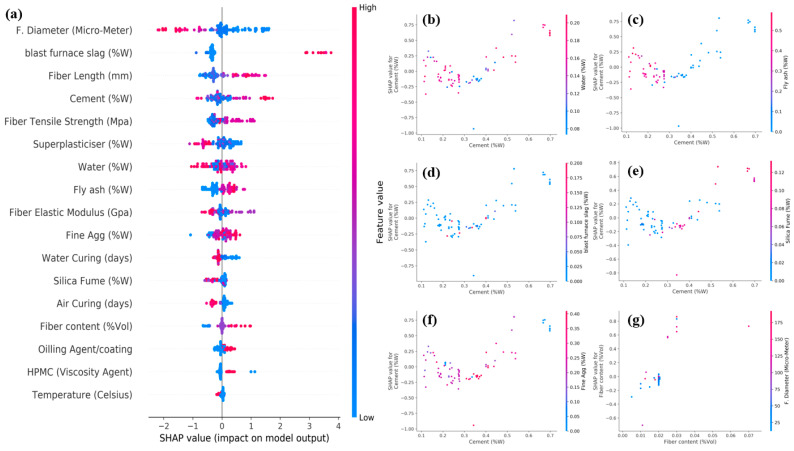
The SHAP analysis showing the influence of each continuous feature on ECC’s tensile strain: (**a**) global feature importance; (**b**) cement SHAP vs. water content; (**c**) cement SHAP vs. fly ash; (**d**) cement SHAP vs. blast furnace slag; (**e**) cement SHAP vs. silica fume; (**f**) cement SHAP vs. fine aggregate; (**g**) interaction of fiber content and length.

The Kernal SHAP analysis on ECC’s tensile strain is depicted in Figure 15. The most important five features included fiber diameter, blast furnace slag, fiber length, cement, and fiber tensile strength, three of which were fiber parameters. High values of fiber diameter had negative SHAP values, which were in accordance with the findings of the PDP analysis. In contrast, the fiber length possessed a similar positive trend to ECC’s tensile stress and strain. Figure 15b–f shows the individual SHAP values of cement with other features as a function of cement content on the x-axis. Cement content was negatively correlated with mortar strain when it was below around 0.3 wt. However, this trend reversed when cement content exceeded 0.3 wt. When the cement content is below a critical threshold, the matrix strength is significantly reduced, which leads to diminished interfacial bonding strength between the fibers and the matrix. At a given fiber content, fibers are prone to completely debond from the matrix under tensile stress, negating strain-hardening behavior. Furthermore, the water content shown in Figure 15b indicates that the water-to-cement ratio should be kept low to achieve a high cement SHAP, which is mainly attributed to a dense mortar matrix. Additionally, the effect of fly ash, blast furnace slag, and fine aggregate on the SHAP value of cement was not apparent compared to that of silica fume, which can be seen in Figure 15c–f. Fiber content was positively correlated with the SHAP value, improving the ECC’s tensile strain, as shown in Figure 15g. This is consistent with the outcome derived in PDPs.

### 4.4. Generalization and Limitations of the Proposed Framework

The generalization capability of the proposed EDFrame is improved by the increased diversity of training data introduced through data augmentation, which enables the model to better capture complex nonlinear relationships among mixture parameters. This is particularly beneficial for ECC datasets with a limited sample size and high feature variability. However, the generalization performance remains inherently constrained by the underlying data distribution, and the model may exhibit reduced reliability when applied to scenarios that fall outside the main data range or involve unseen combinations of input features.

It should be noted that GAN-based augmentation has inherent limitations. Specifically, the generator tends to learn dominant data patterns and may have limited ability to accurately reproduce rare or extreme cases, which are typically underrepresented in the original dataset. In particular, deviations in discrete feature distributions may further influence the reliability of generated samples in extreme cases. As a result, although the augmented data improve overall model performance, prediction uncertainty may still increase in low-density regions of the feature space.

In addition, the dataset compiled from literature sources inevitably introduces heterogeneity due to variations in experimental conditions, material properties, specimen preparation, and reporting standards across different studies. Although strict selection criteria were applied to improve data consistency and reliability, such variability cannot be completely eliminated and may introduce additional uncertainty into the model. Therefore, caution is required when interpreting the results, especially when extending the model to new datasets or different engineering contexts.

## 5. Conclusions

Engineered cementitious composite (ECC) is a strain-hardening cementitious material with a high tensile strength. This study proposes an integrated prediction framework (EDFrame) to tackle three issues: data augmentation, deep learning (DL) prediction, and model interpretability. The main conclusions are drawn as follows:

(1) The proposed Tuned-CTGAN outperformed the GaussianCopula Model and TVAE in data augmentation, as evidenced by its higher KSTest and CSTest values for 18 features. The reliability of virtual data generation by Tuned-CTGAN was verified by the distribution plot, cumulative sum, and correlation table.
(2) Among all the ML and DL models, the RF model and 1D-Residual CNN illustrated the highest prediction accuracy in the original ECC’s tensile stress and strain datasets, respectively. After data augmentation by Tuned-CTGAN, the *R*^2^ of 1D-Residual CNN on stress and strain datasets was significantly increased from 0.8658 and 0.8433 to 0.9128 and 0.9378, respectively, demonstrating the optimal prediction accuracy.
(3) PDP analysis revealed that the optimal fiber content range for tensile stress enhancement is 1.5% to 2%. Additionally, fibers with a length range of 12 mm to 20 mm, a diameter below 50 micro-meters, and an elastic modulus above 300 GPa or below 70 GPa are more likely to produce higher ECC tensile strain.
(4) The mortar compositions comprising cement, water, and silica fume were observed to be the most important features of ECC’s tensile stress. Conversely, the fiber diameter, length, and tensile strength significantly influenced the tensile strain of ECC. Additionally, the cement content of 0.3 wt was the extreme point in the response to the tensile strain.

Based on the above findings, the proposed EDFrame offers practical value for ECC mixture design by enabling rapid and accurate performance prediction prior to laboratory testing. The established 1D-Residual CNN model can serve as a screening tool, allowing users to input candidate mixture parameters and obtain reliable predictions of tensile stress and strain within seconds, thereby reducing trial-and-error experimentation and accelerating the design cycle. The PDP and SHAP analyses provide actionable design guidelines, such as the optimal fiber content range of 1.5–2% and fiber length range of 12–20 mm, directly informing mixture proportioning decisions. Despite these advantages, some limitations should be noted. The generated data may not fully reproduce the distribution of discrete features, and the prediction performance may be constrained in extreme or underrepresented cases. Future work will focus on improving data generation quality and enhancing model robustness to further strengthen generalization capability.

## Figures and Tables

**Table 1:** The statistically based metrics based on three augmentation methods and stress and strain datasets.

Variables and Outcome	Tuned-CTGAN	Gaussian Copula Model	TVAE
Cement (C)	0.8805/0.8914	0.9394/0.9131	0.866/0.8986
Water (C)	0.8144/0.8746	0.9291/0.9134	0.8001/0.7617
Fine Agg (C)	0.8236/0.8773	0.7216/0.7264	0.8623/0.799
Fly ash (C)	0.7887/0.8104	0.5067/0.5689	0.8049/0.8448
Silica Fume (C)	0.8211/0.8014	0.5993/0.6484	0.7943/0.7782
Blast furnace slag (C)	0.7502/0.7892	0.1756/0.1348	0.7878/0.7191
Superplasticiser (C)	0.8551/0.8664	0.8219/0.8465	0.7728/0.8466
Fiber type (D)	0.7108/0.7144	0.0068/0.7647	0.3686/0.0031
Fiber content (C)	0.6752/0.6551	0.6068/0.593	0.7122/0.6765
F. Diameter (C)	0.7884/0.7757	0.6719/0.6848	0.6772/0.5834
F. Length (C)	0.7928/0.7993	0.8131/0.8099	0.7599/0.7697
F. Tensile Strength (C)	0.7877/0.74	0.7638/0.7612	0.7862/0.7908
F. Elastic Modulus (C)	0.8377/0.7557	0.6899/0.7068	0.8269/0.8357
HPMC (C)	0.6902/0.59	0.7457/0.9748	0.6505/0.8099
Oiling Agent (C)	0.8963/0.9215	0.9943/0.9988	0.8539/0.8554
Temperature (C)	0.6618/0.6456	0.5637/0.5605	0.6802/0.7378
Water Curing (C)	0.7/0.7438	0.7067/0.707	0.6922/0.7357
Air Curing (C)	0.7124/0.7207	0.3187/0.3105	0.6883/0.7323
Peak tensile stress (C)	0.8342	0.9016	0.8248
Peak tensile strain (C)	0.8761	0.9309	0.9209
Average KSTest value	0.7784/0.7908	0.6928/0.7105	0.7689/0.7831
Average CSTest value	0.7108/0.7144	0.0068/0.7647	0.3686/0.0031

It is noted that the former values are the metrics for the tensile stress dataset and the latter values are for the tensile strain dataset.

## Data Availability

The original contributions presented in this study are included in the article. Further inquiries can be directed to the corresponding author.

## Appendix A

**Table A1:** Statistical analysis of variables for the original 450 ECC stress data.

Variables	Minimum	Maximum	Mean	Std Dev	Skewness	Kurtosis
Cement (%wt)	0	0.755	0.345	0.158	0.652	−0.168
Water (%wt)	0.027	0.230	0.141	0.044	−0.207	−0.857
Fine Agg (%wt)	0	0.541	0.227	0.120	−0.184	−0.181
Fly ash (%wt)	0	0.646	0.173	0.200	0.600	−1.167
Silica Fume (%wt)	0	0.246	0.047	0.054	0.920	0.200
Blast furnace slag (%wt)	0	0.439	0.039	0.099	2.451	4.446
Superplasticiser (%wt)	0	0.224	0.009	0.013	10.952	181.047
Fiber content (%vol)	0.003	0.200	0.023	0.019	7.550	64.010
F. Diameter (micro-meter)	7.668	625	71.808	103.925	3.829	16.649
F. Length (mm)	2	38	14.227	5.659	0.997	1.533
F. Tensile Strength (MPa)	850	6360	2411	902	0.869	1.858
F. Elastic Modulus (GPa)	6	472	105	75.233	1.927	5.042
HPMC	0	1	0.090	0.280	2.899	6.534
Oiling Agent	0	1	0.254	0.436	1.135	−0.716
Temperature (Celsius)	0	500	35	46	5.657	42.676
Water Curing (days)	0	91	21.140	16.025	1.661	6.006
Air Curing (days)	0	112	5.189	12.818	4.195	25.092
Peak tensile stress (MPa)	1.8	33.4	7.279	4.537	1.838	5.551

**Table A2:** Statistical analysis of variables for the original 400 ECC strain data.

Variables	Minimum	Maximum	Mean	Std Dev	Skewness	Kurtosis
Cement (%wt)	0.093	0.698	0.337	0.161	0.737	−0.166
Water (%wt)	0.029	0.230	0.144	0.044	−0.367	−0.771
Fine Agg (%wt)	0	0.541	0.228	0.125	−0.184	−0.352
Fly ash (%wt)	0	0.646	0.19	0.209	0.417	−1.437
Silica Fume (%wt)	0	0.246	0.041	0.052	0.991	0.293
Blast furnace slag (%wt)	0	0.384	0.028	0.085	3.051	7.829
Superplasticiser (%wt)	0	0.224	0.009	0.013	10.499	164.643
Fiber content (%vol)	0.003	0.200	0.023	0.020	7.179	57.334
F. Diameter (micro-meter)	7.668	320	61.444	59.266	1.988	3.395
F. Length (mm)	2	38	13.546	5.924	1.253	1.836
F. Tensile Strength (MPa)	850	6360	2339	912	1.210	2.309
F. Elastic Modulus (GPa)	6	472	101	79.626	1.881	4.362
HPMC	0	1	0.110	0.310	2.522	4.413
Oiling Agent	0	1	0.283	0.451	0.966	−1.072
Temperature (Celsius)	0	500	35	48	5.567	40.328
Water Curing (days)	0	91	19.363	14.826	1.464	5.516
Air Curing (days)	0	112	7.043	17.164	3.502	13.822
Peak tensile strain (%)	0.017	17.3	3.053	2.620	1.501	2.965
